# A comprehensive dataset on the extraction of pigments from Oscillatoria spp.

**DOI:** 10.1016/j.dib.2023.109972

**Published:** 2023-12-18

**Authors:** Jannatul Nayeem, Proma Dey, Sumit Kanti Dey, Dipa Debi, Mohammed Abdullah Ayoun, Helena Khatoon

**Affiliations:** Department of Aquaculture, Chattogram Veterinary and Animal Sciences University, Chattogram-4225, Bangladesh

**Keywords:** Pigment, Purification factor, Chlorophyll, Phycobiliprotein

## Abstract

Cyanobacterial species such as *Oscillatoria* spp. pigments are essential components that enable the photosynthetic ability of this autotrophic organism. These pigments, principally chlorophylls and phycobiliproteins, are crucial for photosynthesis and give cyanobacteria their distinctive blue-green color. Exploring these pigments is crucial for unraveling the ecological and biotechnological relevance and significance. Spectrophotometric methods were used for measuring the chlorophyll-a, phycobiliprotein, and carotenoid contents of *Oscillatoria* species. *Oscillatoria* spp. displayed significantly variable (*p* ˂ 0.05) chlorophyll-a ranging from 12.67 ± 0.04 to 22.72 ± 0.04 µg/mL. Phycobiliprotein content (mg/g) significantly (*p* ˂ 0.05) varied from 87.39 ± 0.12 µg/mL to 121.42 ± 0.06. Carotenoid content also significantly ranged from 1.0 ± 0.01 µg/mL to 1.4 ± 0.01 µg/mL. Present data will contribute to the screening and characterization of *Oscillatoria* spp. in terms of pigment to utilize it in rigorous scientific research and diverse commercial applications.

Specifications Table

**Table:** 

Subject	Food Science, Aquatic Science
Specific subject area	Chlorophyll-a, phycobiliprotein, purification factor and carotenoid contents of indigenous Oscillatoria spp.
Data format	Raw and analyzed primary data
Type of data	Graph, Table and Picture
Data collection	Data on chlorophyll-a content, phycobiliprotein, purity and carotenoid content were attained by spectrophotometric analysis. For chlorophyll-a content: spectrophotometric analysis at 750 nm, 664 nm, 647 nm, and 630 nm wavelengths. For phycobiliprotein: Phycocyanin, allophycocyanin, phycoerythrin (562 nm, 615 nm, 652 nm, and 720 nm) For purification factor: Purity of Phycocyanin, allophycocyanin, phycoerythrin (280 nm, 565 nm, 620 nm, 650 nm) For carotenoid: spectrophotometric analysis at 450 nm absorbance. The acquired data were further analyzed through MS Excel and IBM SPSS (v. 26.0) software.
Data source location	Disease and Microbiology laboratory, Department of Aquaculture, Chattogram Veterinary and Animal Sciences University (CVASU), Khulshi-4225, Chattogram, Bangladesh
Data accessibility	Data are available with this article and also at Repository name: Mendeley Data Data identification number: DOI:10.17632/tchp9jznkb.2 Direct URL to data: https://data.mendeley.com/datasets/tchp9jznkb/1

## 1 Value of the Data

• The findings on pigments could be helpful in screening potential *Oscillatoria* spp. to select species with higher photosynthetic efficiencies, characteristic natural blue or greenish blue color to improve the coloration of skin, shell, feather of ornamental fish, crustaceans and poultry products through incorporation of extracted pigment as feed additive.
• These pigments absorb light energy and facilitating the conversion of pollutants through enzymatic reactions like a catalyst to provide bioremediation; pigments also aid the antioxidant properties, and molecular fluorescent marker potentialities of *Oscillatoria* spp.
• These data enable researchers to outline the insights of primary productivity (photosynthetic activity, species diversity, environment and ecosystem productivity) and physiological mechanisms of *Oscillatoria* sp. such as broader light absorption range, photoprotective compounds production.
• Pigment data also facilitates the extraction of valuable pigments which are utilized as natural alternatives to synthetic colorants in food, cosmetics and pharmaceuticals. These data encourage innovation and sustainability in commercial as well as in research sectors.

## 2 Data Description

Pigment contents such as chlorophyll, carotenoid, and phycobiliproteins were evaluated and presented in this dataset [1]. Amounts of chlorophyll-a, and carotenoid content were measured in µg/mL, while the phycobiliprotein contents were measured in mg/g. Chlorophyll-a content, primary pigment of *Oscillatoria* sp. involved in oxygenic photosynthesis as well as converting absorbed photon energy to chemical energy which directly influence the productivity, diversity, distribution etc. of *Oscillatoria* sp.; carotenoid data also complement the chlorophyll data to provide antioxidant properties and safeguard *Oscillatoria* cells in excessive light. Phycobiliproteins act as accessory pigments of *Oscillatoria* sp. that capture broad spectrum of blue and red light for photosynthesis. Higher phycobiliprotein data enable the selectivity of diversified species with higher photosynthetic ability, adaptive strategies, resilience to environmental changes. Thus, pigment data support overall primary productivity of *Oscillatoria* sp.

**Fig. 1:**
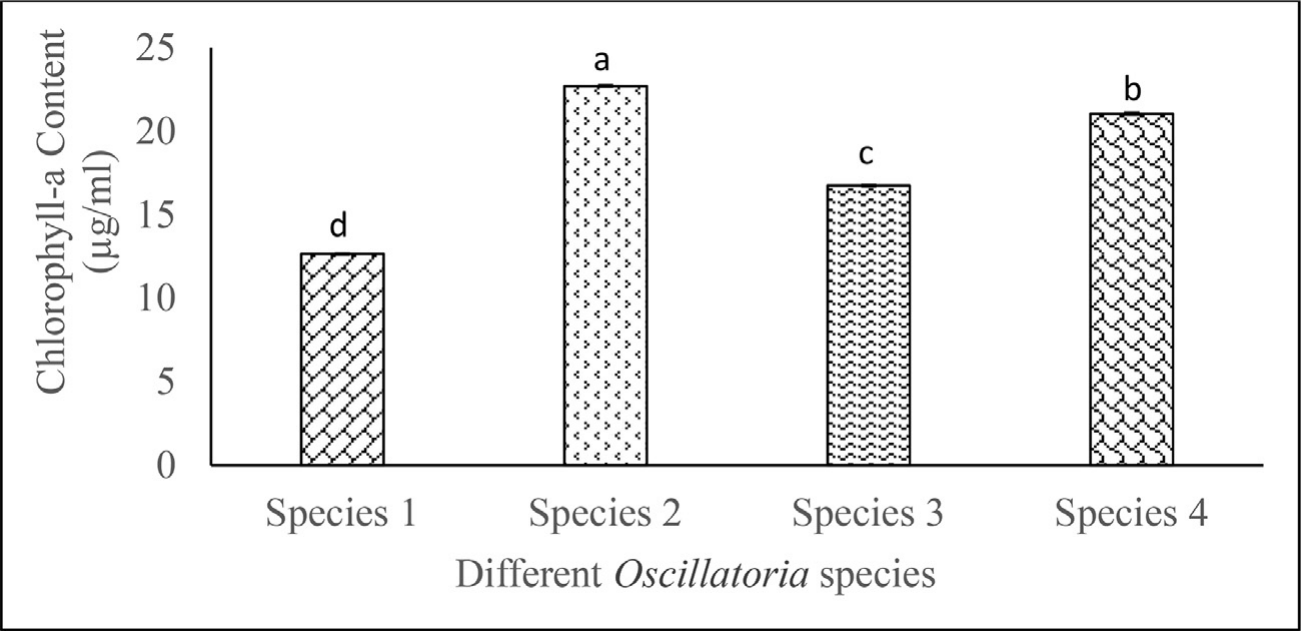
Chlorophyll-a content (means ± SE) of isolated marine and freshwater *Oscillatoria* species. Significant variations among the species (*p* < 0.05) are denoted by values in each category with a distinct letter.Significant variations of the phycocyanin, allophycocyanin, phycoerythrin content of the *Oscillatoria* spp. were obtained and presented in Table 1. Phycobiliprotein contents also showed significant variations (*p* < 0.05) among the *Oscillatoria* species.

**Fig. 2:**
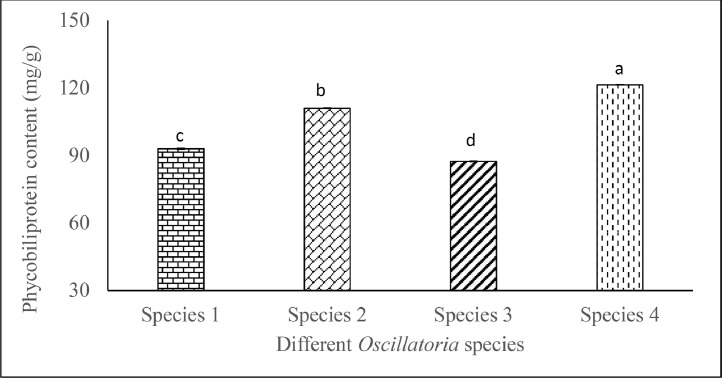
Total phycobiliprotein content (mg/g) of Oscillatoria spp. Values are represented as the average of the triplicates with standard error (mean ± SE). Different letters within each value series indicate significant differences (*p* < 0.05) among the species.

*Oscillatoria* exhibit significant chlorophyll content variation in Fig. 1. Significantly highest (*p* < 0.05) chlorophyll-a content (22.72 ± 0.04 µg/mL) was found in *Oscillatoria* sp. 2, whereas *Oscillatoria* sp. 1 showed significantly lower (*p* < 0.05) amount of chlorophyll-a content (12.67 ± 0.04 µg/mL) (Fig. 2).

**Table 1:** Phycocyanin, allophycocyanin, phycoerythrin content (mg/g) of Oscillatoria spp. Values are presented as mean of the triplicates with standard error bar (SE = σ/√n). Significant variations among the species (*p* < 0.05) are indicated by values in each series with a distinct letter.

Phycobiliprotein Extracts (mg/g)	*Oscillatoria*			
	Species 1	Species 2	Species 3	Species 4
Phycocyanin	81.85 ± 0.84^c^	84.75 ± 0.12^b^	69.00 ± 0.12^d^	93.47 ± 0.08^a^
Allophycocyanin	10.78 ± 1.00^b^	18.19 ± 0.06^a^	17.00 ± 0.12^a^	16.13 ± 0.12^a^
Phycoerythrin	0.36 ± 0.14^d^	8.10 ± 0.15^b^	1.40 ± 0.21^c^	11.82 ± 0.14^a^

**Fig. 3:**
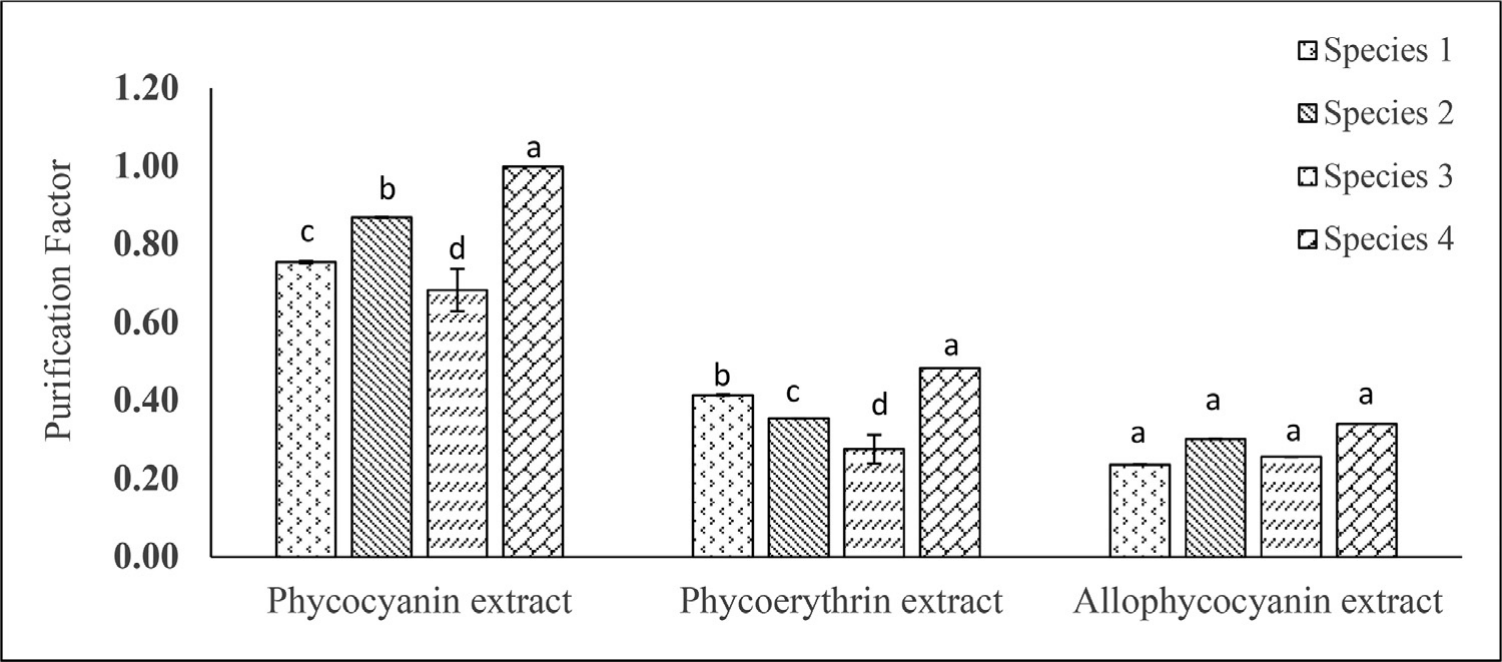
Purity of phycocyanin, phycoerythrin and allophycocyanin raw extracts of *Oscillatoria* species. Significant variations among the species (*p* < 0.05) are indicated by values in each series with a distinct letter.

The purification factor of phycocyanin, phycoerythrin and allophycocyanin raw extract of *Oscillatoria* sp. is presented in Fig. 3 where highest and lowest significant (*p* < 0.05) phycocyanin, phycoerythrin and allophycocyanin obtained from species 4 (1.00, 0.48, 0.34) and species 3 (0.68, 0.28, 0.26) respectively. There are no significant (*p* >0.05) variations in the purity of allophycocyanin raw extracts (*p* = 0.188) among the species.

**Fig. 4:**
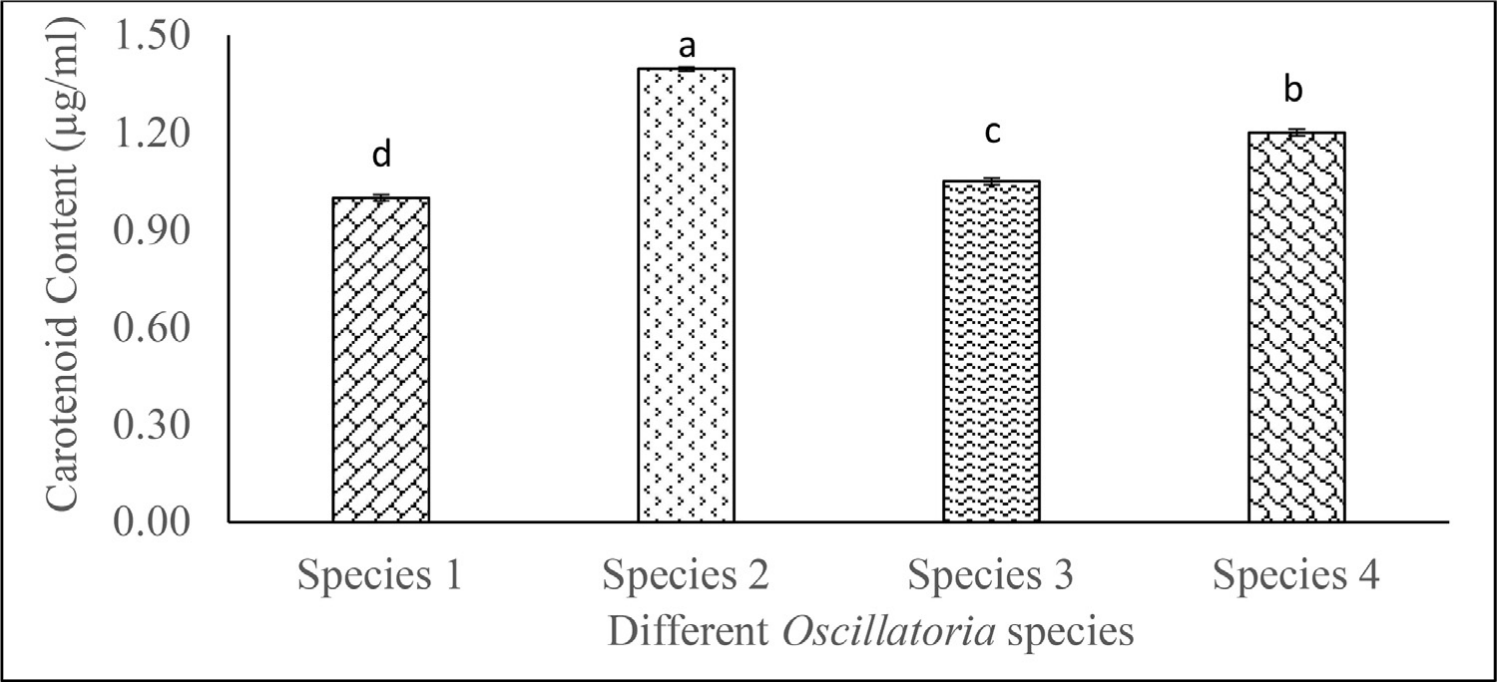
Carotenoid content (means ± SE) of isolated *Oscillatoria* spp. Distinct letter in each category of value indicate significant variations among the species (*p* < 0.05).

Carotenoid content of *Oscillatoria* spp. varied from 1.00 ± 0.01 µg/mL to 1.4 ± 0.01 µg/mL (Fig. 4). Statistically significant (*p* < 0.05) differences were analyzed among the *Oscillatoria* spp. where highest and lowest significant (*p* <0.05) carotenoid content was obtained from *Oscillatoria* sp. 2 and *Oscillatoria* sp. 1 respectively.

## 3 Experimental Design, Materials and Methods

### 3.1 Culture of Oscillatoria spp

#### 3.1.1 Collection and culture of pure isolates

**Fig. 5:**
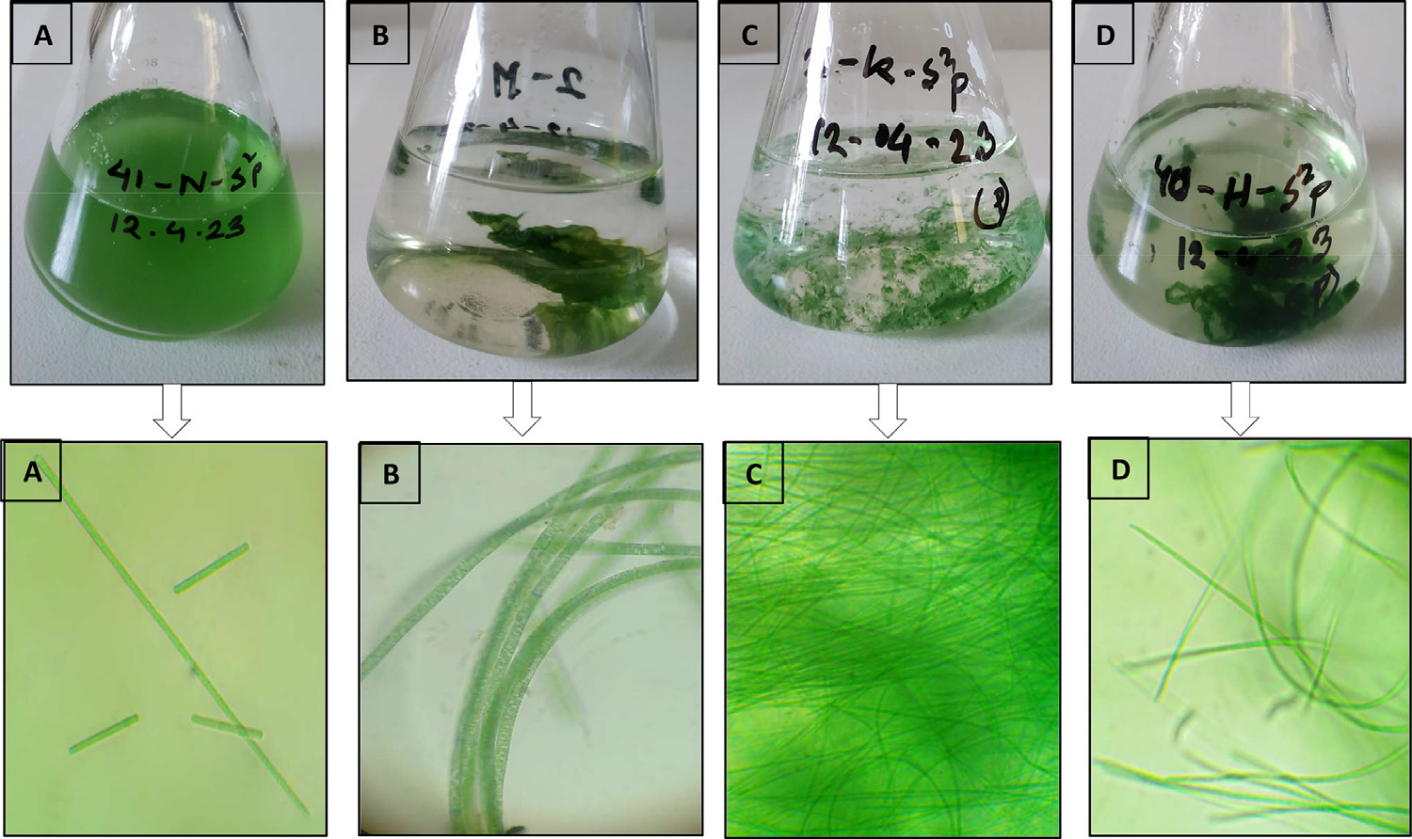
Colony structures and microscopic view of *Oscillatoria* sp. 1 (A- planktonic), *Oscillatoria* sp. 2 (B- highly filamentous), *Oscillatoria* sp. 3 (C- moderate filamentous), *Oscillatoria* sp. 4 (D-highly filamentous). A, B represent the marine and C, D represent the freshwater *Oscillatoria* species.

Pure isolates of two freshwater and two marine water *Oscillatoria* sp. were obtained from Live Feed Culture laboratory of Department of Aquaculture, Faculty of Fisheries, Chattogram Veterinary and Animal Sciences University, Chattogram, Bangladesh. Pure isolates were inoculated and cultured in the Bold's Basal Medium (BBM) [2] and Conway medium [3] respectively. For stock culture maintenance, 100 ml inoculum of *Oscillatoria* sp. was inoculated into 900 ml of respective culture medium. Then the cultures of *Oscillatoria* sp. 1 (planktonic; marine), *Oscillatoria* sp. 2 (highly filamentous; marine), *Oscillatoria* sp. 3 (moderate filamentous; freshwater), *Oscillatoria* sp. 4 (highly filamentous; freshwater) were incubated in controlled indoor condition at 24 °C temperature for 13 days, 10 days, 11 days, 9 days respectively. The cultures were maintained under continuous light conditions of 12 h light: 12 h dark regime, with 2000 lux light intensity [4] (Fig. 5).

#### 3.1.2 Mass culture in plastic jars

Filtered and autoclaved (121 °C and 15 lbs. /inch^2^ pressure for 15 mins) distilled water and sea water were used to prepare BBM and Conway media for mass culture sequentially. Growth media were prepared using required nutrient solutions; in case of marine stocks, salinity was maintained according to the salinity found in its sampling site. Marine stocks such as *Oscillatoria* sp. 1 and *Oscillatoria* sp. 2 were cultured in Conway medium with maintained salinities of 20 ppt and 25 ppt respectively. Pure stocks were then transferred to 20 L plastic tanks for mass culture at 25 °C with a photoperiod of 12 h:12 h (light: dark), commencing with a 5 L volume and eventually scaling up to around 16 L to optimize growth conditions through proper adaptation. Every two days, media was added to the culture tank until it reached the desired volume. In mass culture, initiating from smaller quantity ensures precise control to promote healthy initial growth. Gradual volume expansion avoids sudden culture shocks and aids *Oscillatoria* sp. adaptation for consistent growth and reduced contamination risks. Economically, it conserves resources, allowing adjustments and steady nutrient supply for sustained conditions throughout the mass culture. Concurrently, individual culture tanks were continuously aerated using a centralized air pump. PVC pipe substrates were used for *Oscillatoria* sp. mass culture and samples from each tank were examined under a light microscope weekly to check the purity of the stock.

### 3.2 Harvesting and dried biomass of Oscillatoria sp

After respective mass cultures, 500 ml of liquid *Oscillatoria* spp. biomass were meticulously dispensed into each of four centrifuge jars with maximum capacity of 750 mL and then harvested by centrifugation at 5000 rpm for 5 min by using centrifugation machine (TL5R Free Standing low speed refrigerated centrifuge, Herexi). The wet microalgae biomass obtained from post-centrifugation was subsequently oven-dried overnight at 40 °C through a high-quality hot air oven (JSR Korea's Natural Convention Oven LNO-150) and the dried biomass was crushed into fine powders by using a mortar and pestle. The powdered microalgae were then stored in a standard freezer at 4 °C until required for further use.

### 3.3 Determination of chlorophyll

#### 3.3.1 Extraction of Oscillatoria sp

Extraction of *Oscillatoria* sp. was perform using chemical method. To extract *Oscillatoria* spp. for chlorophyll content, 1 ml MgCO_3_ was filtered through a filter paper (47 mm Ø Whatman® GF/C glass microfiber) using a filter machine. Following this, 1 ml of each algae sample was filtered and the edges of filter paper containing no residue were trimmed. The filter paper was then folded and placed in a 15 ml centrifuge tube with the center facing downwards. Then, 2 ml of 90% acetone was added and ground for 1 min, followed by the addition of 8 ml of 90% acetone and grinding for 30 s. The sample was then refrigerated in the dark for 1 h. After 1 h, the sample was centrifuged at 3000 rpm for 10 min, and the acetone extract was transferred to another centrifuge tube and centrifuged at low speed (500 rpm) for 5 min. Finally, the absorbance of the acetone extract was measured against 90% acetone as a blank.

#### 3.3.2 Chlorophyll quantification

Chlorophyll concentration was quantified based on spectrophotometric method [5]. The clear acetone extract was carefully transferred into a 1 cm cuvette and optical density (OD) was recorded at 750 nm, 664 nm, 647 nm, and 630 nm wavelengths. The OD values at 664 nm, 647 nm, and 630 nm were used to calculate chlorophyll concentration, while the OD value of 750 nm was used as turbidity correction factor and subtracted from each of the pigments OD values before using them in the equations. The concentrations of chlorophyll a, was calculated using the corrected OD values in the following equations [6]:

$$c_{a}=11.85\left(\text{OD}664\right)-1.54\left(\text{OD}647\right)-0.08\left(\text{OD}630\right)$$

Where: C_a_= Chlorophyll-a concentration in mg/L, and OD664, OD647, and OD630 = corrected optical densities (with a 1 cm light path) at the respective wavelengths. Once the pigment concentrations in the extract were determined, the pigments' quantity per unit volume was computed using the following formula:

$$\text{Chlorophyll}\;a\;\left(\text{mg}/m^{3}\right)=\frac{\text{Ca}\;\left(\text{mg}/L\;\right)\times \text{extract}\;\text{volume}\;\left(L\right)}{\text{volume}\;\text{of}\;\text{sample}\;\left(m^{3}\right)\;}$$

Finally, the Chlorophyll a content unit was converted from mg/m³ to µg/mL to standardize the chlorophyll-a unit in *Oscillatoria* spp. This adjustment accommodates small sample volumes and ensures alignment with micromolar concentrations within cellular structures.

### 3.4 Phycobiliproteins determination

Spectrophotometric method was used to estimate the phycobiliproteins in Cyanobacteria [7]. 40 mg dried powder was mixed with 10 ml phosphate buffer (pH 7.0; 0.1 M) using vortex mixture and stored for 24 h at 4 °C. Then, the samples were centrifuged at 6000 rpm for 10 min. Finally, the supernatant was collected and absorbance of the samples was measured against the phosphate buffer solution as blank at specific wavelengths (562 nm, 615 nm, 652 nm, and 720 nm) using a spectrophotometer (Nano Drop Spectrophotometer, Model-Nanoplus, Germany). The absorbance at 720 nm was used to measure the cellular debris. The amount of phycocyanin (PC), allophycocyanin (APC) in the sample was calculated from the absorbance [8] and phycoerythrin (PE) was also calculated [7] using the following formula:

$$\text{Phycocyanin}\;\left(\text{PC}\right)\;\text{mg}/\text{mL}=\left\{A_{615}-A_{720})-0.474x\left(A_{652}-A_{720}\right)\right\}/5.34$$

$$\text{Allophycocyanin}\;\left(\text{APC}\right)\;\text{mg}/\text{mL}=\left\{A_{652}-A_{720})-0.208x\left(A_{615}-A_{720}\right)\right\}/5.09$$

$$\text{Phycoerythrin}\;\left(\text{PE}\right)\;\text{mg}/\text{mL}=\left\{A_{562}-\left(2.41x\text{PC}\right)-\left(0.849x\text{APC}\right)\right\}/9.62$$

Total phycocyanin, phycoerythrin, and allophycocyanin (mg/g) were calculated according to [9] as follows:

P=(Pigmentconcentration×V)/DB

Where, V= Solvent volume, DB= Dried biomass

Total phycobiliproteins (mg/g) were further estimated from the count of the phycocyanin, phycoerythrin, and allophycocyanin contents in dried microalgae biomass.

### 3.5 Purification factor

Purification factor or purity ratio indicates the level of impurities present in the extracted compound. The commercial value of phycobiliproteins is greatly influenced by their purity grade. Higher purity ratios signify a greater concentration of the desired phycobiliprotein with fewer contaminants or other proteins. Phycobiliprotein purity more than 0.7 is regarded as food grade, purity over 1.5 as cosmetic grade, more than 3.9 as reactive grade and above 4.0 as an analytical grade [10] The purification factor of phycocyanin, phycoerythrin, and allophycocyanin extract was determined spectrophotometrically by A 620/A 280, A 565/A 280 and A 650/A 280 ratio [8].

### 3.6 Determination of carotenoids

Carotenoids were determined from the wet algal suspension [11]. 1 mL algal sample from each mass culture was collected at their stationary phase of growth curve. Collected sample was centrifuged at 1000 g for 5 mins. Following this, the obtained pellet was extracted with 3 mL of ethanol: hexane (2:1) (v/v). Then, 2 mL water and 4 mL hexane (Sigma, USA) were added, vigorously shaken and again centrifuged at 1000 × *g* for 5 mins. Finally, absorbance of the isolated hexane layer was determined at 450 nm wavelength through spectrophotometer. The amount of extracted carotenoids from the samples in micrograms were determined by multiplying 25.2 with absorbance (A_450)_ [12].

### 3.7 Statistical analysis

IBM SPSS (v. 26.0) was used for statistical analyses regarding the chlorophyll-a, phycobiliproteins, purification factor, carotenoid contents. Descriptive statistics were performed for each of the parameters for each *Oscillatoria* sp.; following that, a test for homogeneity of variance was also performed to make valid comparisons among the *Oscillatoria* species. It also provides precise estimation of statistical parameters which ensures the accuracy, validity, and reliability of statistical analyses and conclusions drawn from those analyses. Collected data were analyzed by one-way analysis of variance (ANOVA) and significant differences amongst *Oscillatoria* species were analyzed through Tukey's multiple comparison tests at 95% confidence interval level. To discern differences between groups, post-hoc test was utilized.

## CRediT authorship contribution statement

**Jannatul Nayeem:** Methodology, Data curation, Writing – original draft. **Proma Dey:** Data curation, Formal analysis. **Sumit Kanti Dey:** Data curation. **Dipa Debi:** Data curation, Formal analysis. **Mohammed Abdullah Ayoun:** Data curation, Formal analysis. **Helena Khatoon:** Conceptualization, Funding acquisition, Supervision, Resources, Validation, Writing – review & editing.

## Data Availability

A comprehensive dataset on the extraction of pigments from Oscillatoria spp. (Original data) (Mendeley Data).
